# EEG Functional Connectivity Underlying Emotional Valance and Arousal Using Minimum Spanning Trees

**DOI:** 10.3389/fnins.2020.00355

**Published:** 2020-05-07

**Authors:** Rui Cao, Yan Hao, Xin Wang, Yuan Gao, Huiyu Shi, Shoujun Huo, Bin Wang, Hao Guo, Jie Xiang

**Affiliations:** ^1^College of Software Engineering, Taiyuan University of Technology, Taiyuan, China; ^2^College of Computer Science and Technology, Taiyuan University of Technology, Taiyuan, China

**Keywords:** emotion, negative bias, functional connectivity, graph theory, minimum spanning tree

## Abstract

In recent years, traditional methods such as power spectrum and amplitude analysis have been used to research the emotional electroencephalogram (EEG). The brain network method is also used in emotional EEG research, which can better reflect the activity of brains. A minimum spanning tree (MST) represents the key information flow in the weighted brain network, and it provides a sensitive method to capture subtle information in network organization while effectively avoiding the shortcomings of traditional brain networks. The DEAP dataset provides electroencephalogram (EEG) data for four categories of emotions: high arousal and high valence (HAHV), high arousal and low valence (HALV), low arousal and high valence (LAHV), and low arousal and low valence (LALV). Phase lag index (PLI) weighted matrices were calculated in five frequency bands. On this basis, the minimum spanning trees were constructed. At the same valence level in the gamma (γ) band, HAHV and HALV showed significant higher mean PLI (MPLI), maximum degree (Degree_max_) and leaf fraction and significant lower diameter and eccentricity than LAHV and LALV. At the same arousal level in the γ band, HALV showed significant higher MPLI, Degree_max_ and leaf fraction and significant lower diameter and eccentricity than HAHV. These results indicate that the low-arousal showed more line-shaped configurations than the high-arousal. Additionally, in the high-arousal condition, a shift toward more star-shaped trees from high-valence to low-valence supports the trend toward randomness of the brain network with negative emotions and that the brain is more activated when faced with negative emotions. From a brain network perspective, this phenomenon provides a theoretical basis for negative bias.

## Introduction

Emotion processing is one of the advanced cognitive functions of the human brain. Increasing attention has been paid to the study of the influence of different emotions on the human brain. Electroencephalogram (EEG) and event-related potential (ERP) recording can capture and assess neural responses to affective events with a high temporal resolution and are widely used in emotional research. In general, affective content can produce stronger emotional effects than neutral content can (Olofsson et al., 2008). Many studies have demonstrated this using synchronicity (Zhang et al., 2012), amplitude (Pegg et al., 2019) and other characteristics. Furthermore, some studies find that bad moods have a larger impact than good moods and that events involving unpleasant emotions remain more salient in people’s minds than events involving pleasant emotions do (Baumeister et al., 2001; Amrisha et al., 2008). When studying emotional facial expression, Li found that the coherence of negative emotion was greater than that of positive emotion in both the low and high γ bands (Li et al., 2015). Zhu L found that the phase lock value of positive video stimulation was significantly lower than that of negative stimulation in the β and γ bands (Zhu et al., 2018).

At present, traditional EEG analysis methods, including methods that focus on the power spectrum (Liu et al., 2018), coherence (Zhang et al., 2012; Mu et al., 2017) and phase lock value (Zhu et al., 2018), are widely used. The brain regions coordinate and cooperate with each other to form a complex brain network, and the development of graph theory provides a perfect tool for brain network analysis (Rubinov and Sporns, 2010; Calhoun et al., 2018). Graph theory can sensitively capture subtle changes in the network and has revealed fundamental mechanisms of functional brain organization in EEG analysis (Fraschini et al., 2016). Li used traditional graph theory to find that the number of active brain network connections during negative stimuli was greater than the number of active connections during positive stimuli (Li et al., 2015). These results concerning topological structure provided new evidence that healthy controls had a negative bias in terms of the functional connectivity of brain networks.

Although traditional graph theory provides a new perspective and means of discovery in brain network analysis, the methodology used in traditional graph theory has some problems that cause the same research conditions to result in different—or even opposite—conclusions (Tijms et al., 2013; van Diessen et al., 2015). In particular, a traditional unweighted network involves threshold selection but provides no reasonable way to select the threshold. Improper threshold selection may lead to false links or cause the loss of important information (van Wijk et al., 2010). Even the use of weighted rather than unweighted graphs does not provide an optimal solution because measures computed on these graphs are influenced by the large number of noisy connections and by the average functional connectivity strength (Tewarie et al., 2014). However, a minimum spanning tree (MST) effectively avoids the problems of weighted and unweighted brain networks (Eric et al., 2013; Tewarie et al., 2015). An MST connects all nodes in the network without forming a cycle, and it generates the strongest subconnection. The MST represents the key information flow in the weighted network (it includes the high-probability connections of all the shortest paths in the network) (van Dellen et al., 2014). In recent years, scholars have applied the MST method to study epilepsy (van Diessen et al., 2016), children’s brain development (Boersma et al., 2013), motor imagination (Demuru et al., 2013) and other fields. Moreover, compared with traditional network methods, the MST method has superior sensitivity to small differences in the brain network (Demuru et al., 2013; van Diessen et al., 2016), providing a new tool for research on complex brain networks. To date, there have been few reports on using an MST to analyze the differences between different emotions. The MST method is used in this study to explore the differences in brain network topology when subjects are in different emotional states.

## Materials and Methods

### EEG Data Acquisition

All of the subjects comes from the Dataset for Emotion Analysis using the Physiological and Audiovisual Signals (DEAP) database. The DEAP database is an open database for emotion recognition research based on physiological signals (Koelstra et al., 2012). The database contains EEG data collected from 32 subjects as they watched 40 music videos. These videos had obvious emotional stimulation effects and the duration of each video was 1 min.

### Data Preprocessing

The EEG data were acquired from the DEAP data collection website. The raw EEG data were preprocessed in a series of steps that included down-sampling to 128 Hz, electro-oculogram (EOG) removal and filtering at 4.0–45.0 Hz. The length of each sampling period was 63 s and the first 3 s was a baseline period, which was followed by 60 s of EEG data collected as the subject watched a video.

Each video has two VAD (valence, arousal, dominance) values, one from the behavioral experiment and the other provided by the subjects during the EEG acquisition: while they watched the video, the participants evaluated the video in each VAD dimension using a 9-point scale according to their emotional experiences. Arousal ranged from inactive (e.g., uninterested, bored) to active (e.g., alert, excited), whereas valence ranged from unpleasant (e.g., sad, stressed) to pleasant (e.g., happy, elated). Based on the A and V values, these videos were divided into four types: high arousal/high valence (HAHV) meaning happy and excited, low arousal/high valence (LAHV) meaning calm and chill, low arousal/low valence (LALV) meaning sad and depressed and high arousal/low valence (HALV) meaning angry and shocked (Koelstra et al., 2012).

**FIGURE 1:**
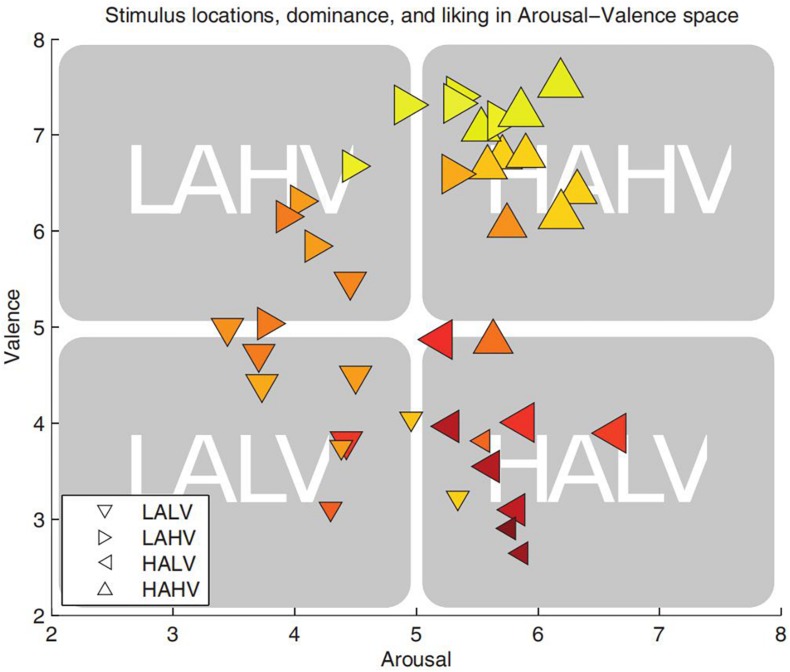
Arousal–valence plane. The valence dimension evaluates the degree of positivity or negativity of the emotion; the arousal dimension describes the intensity of activation associated with the emotion. Liking is encoded by color: dark red is low liking and bright yellow is high liking. Dominance is encoded by symbol size: small symbols stand for low dominance and large for high dominance (Koelstra et al., 2012).

After the online behavior experiment, 40 music videos were selected from 120 videos because the AV value of these 40 videos was more extreme, showing that they provided a better stimulation for emotion (Figure 1). However, a video scoring near one of the coordinate axes could not be accurately classified into one of the selected emotion categories. Thus, in this experiment (Kuai et al., 2017), scores of 1–4 were used as low scores, and scores of 6–9 were used as high scores. In the scoring process, subject number 23 divided the 40 music videos into three categories instead of four, so the EEG data recorded by subject 23 was abandoned and only the remaining subjects’ EEG data were retained.

### Frequency Division

In the human brain, different neuron oscillation frequencies are closely related to the functional activity of the brain (Fellinger et al., 2011; Groppe et al., 2013). Therefore, EEG signals are often divided into different frequency bands in EEG research. In this experiment, EEG data are analyzed in the following five frequency bands: θ (4–7 Hz), α (7–13 Hz), β1 (13–20 Hz), β2 (20–30 Hz) and γ (31–45 Hz).

### Constructing a Correlation Matrix and MST

A network consists of nodes and edges. In this experiment, 32 scalp electrodes were used as the nodes of the network, and the phase lag index (PLI) was used for the network edges. The PLI measures the asymmetry of the distribution of phase differences between two signals, and it is relatively insensitive to the confounding effects of volume conduction (Stam et al., 2014). PLI can reflect the consistency with which one signal’s phase leads or lags relative to another signal, and it is an effective estimation of phase synchronization (Stam et al., 2007). A 32 × 32 correlation matrix can be obtained by calculating the PLI value between each pair of nodes. Whole-brain mean PLI (MPLI) was computed by averaging all the pairwise PLI values, resulting in a single PLI value in order to describe the average functional connectivity for each brain network. Similar methods have been used by Groppe et al. (2013), and the mean synchronization likelihood (SL) was used to characterize the average connection strength of the brain during different emotional stimuli.

For each subject, EEG data was recorded when watching a music video and a PLI matrix was calculated in one frequency band. Then the minimum spanning tree was generated using a Kruskal algorithm (Kruskal, 1956). This procedure started with ranking all connection weights from lowest weight to highest weight. Because we were interested in the strongest connections, we ranked all connections from highest to lowest weight and started by disconnecting all nodes, then added the connection with the highest weight. Next, the connection with the second highest weight was added, and this procedure was repeated until all nodes were connected and it did not form a closed loop. The final MST for each affective brain network contains 32 nodes and 31 edges.

### MST Network Metrics

**FIGURE 2:**
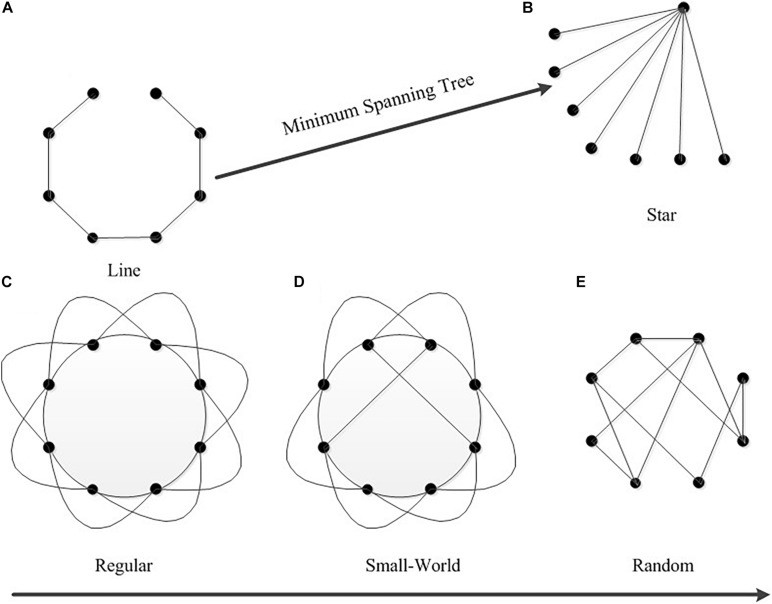
The network structures corresponding to MST topology. Minimum spanning tree topology from a line-shaped to a star-shaped corresponding brain network from regularization to randomization. Panel **(A)** shows a line-shaped MST. Panel **(B)** shows a star-shaped MST. Panel **(C)** shows a regular network, panel **(D)** shows a small world network, and panel **(E)** shows a random network.

The commonly used characteristics of MST analysis include degree, betweenness centrality (BC), eccentricity, diameter, leaf fraction and tree hierarchy, among which degree, BC and eccentricity are local attribute values of individual nodes (Table 1). In order to find the eccentricity, the mean value over all nodes was calculated. The maximum values of BC and Degree (BC_max_ and Degree_max_, respectively) are listed separately. All of the other metrics are global measurements - they characterize the MST as a whole. An important aspect of complex networks is efficient communication between all nodes, which requires a small diameter, i.e., a tree network with a star-shaped topology. In a star-shaped tree, the central node might easily become overloaded because it has a high BC. An optimal tree configuration should therefore strike a balance between diameter reduction and overload prevention (Figure 2). A tree hierarchy measure that captures this trade-off was calculated (Boersma et al., 2013).

**TABLE 1:** MST descriptive statistics.

**Symbol**	**Concept**	**Explanation**	**Formula**
N	Nodes	Number of nodes in MST	–
M	Links	Number of links in the MST	–
*ki*	Degree	Number of links for a given node. Degree_max_ represents the maximum of all node degrees. It may be considered a feature of “hubs,” i.e., crucial regions in the functional brain network.	*ki* = _∑*j* ∈ *N*_*aij*
*Lf*	Leaf fraction	Measured based on the leaf number (the number of nodes that have only one connection). In the formula, L represents the total number of leaves of an MST. When the leaf fraction is high, communication is largely dependent on hub nodes.	*Lf* = *L*/*M*
*D*	Diameter	A measure of the efficiency of global network organization. In the formula, d represents the maximum path length in an MST. In a network with a small diameter, information is efficiently processed between remote brain regions.	D = *d*/*M*
*E*	Eccentricity	The longest optimal path from a reference node to any other node in the MST, where *d*(*i*,*j*) represents the optimal (shortest) path between node *i* and node *j*. The average eccentricity represents the average value of the eccentricity of all nodes. Low average eccentricity means that the nodes of the MST are closer to the hub nodes.	*Ei* = *max*⁡{*d*(*i*,*j*)|*j* ∈ *N*}
BC	Betweenness centrality	Fraction of all shortest paths that pass through a particular node.ρ*hj*is the number of shortest paths between h and *j*, and ρ*hj*^(*i*)^ is the number of shortest paths between h and j that pass through i. BC_max_ represents the maximum BC value of all nodes of an MST. It describes the importance of the most central node, which is a measure of central network organization.	$\text{BC}\,i=\frac{1}{\left(n-1\right)\,\left(n-2\right)}\,\sum_{\begin{array}{c} h,j\in N\\ h\neq i,j\neq i \end{array}}\frac{\rho \,h\,j^{\left(i\right)}}{\rho \,h\,j}$
*Th*	Tree hierarchy	A hierarchical metric that quantifies the trade-off between large scale integration in the MST and central node overloading.	$T\,h=\frac{L}{2\,\text{MBC}\,\max}$

The MST can be regarded as the backbone of the brain network and any change in the topological structure of the MST also sensitively reflects change trends in the brain network. An MST with a small degree, small BC, long diameter and few leaves tends to be “line”-shaped. On the contrary, an MST with a large degree, large BC, short diameter and many leaves tends to be “star”-shaped. Figure 2 shows the network structures corresponding to MST topology. Part A shows an MST with a “line” shape where every node except two end nodes is connected to its two neighbors (low leaf number), but it takes 7 steps to reach the other end of the network (high diameter), and the corresponding network is a regular network. Part B is a “star”-shaped MST which consists of a central node that is connected to other nodes (high degree and BC), which are all leaf nodes (high leaf number). This MST is highly efficient (low diameter) and the corresponding network is a random network. The lower part of Figure 2 shows the underlying topologies of a regular network, a small world network and a random network, which correspond to MSTs from “line” to “star.” As the topological shape changes from a regular network to a random network, the MST diameter and leaf fraction change according to the path length of the underlying network (the diameter of MST is positively related to the path length, and the leaf fraction of an MST is negatively related to the path length).

## Results

**FIGURE 3:**
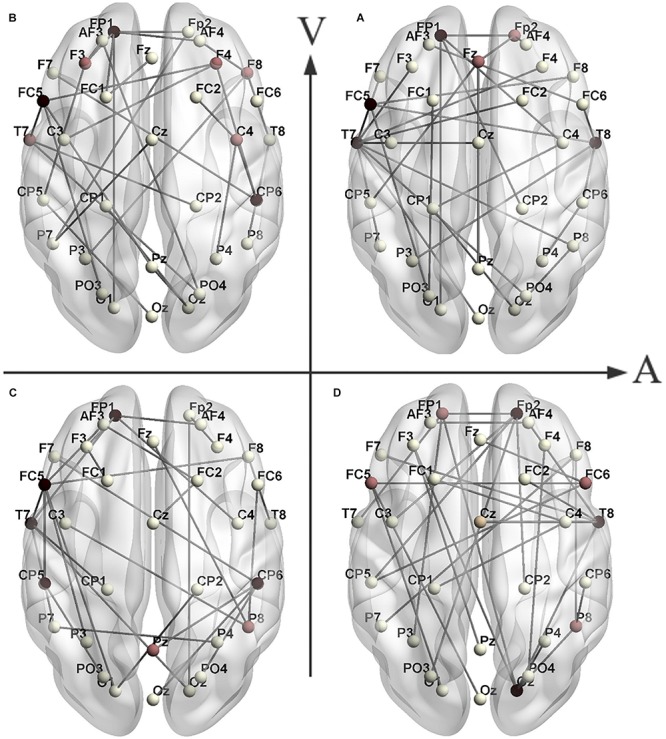
The topological structures of the MST brain networks of the HAHV **(A)**, LAHV **(B)**, LALV **(C)** and HALV **(D)** in the γ band. The horizontal axis represents arousal, and the vertical axis represents valence. Black nodes indicate that the node degree is greater than or equal to 4 and dark red nodes indicate that the node degree is 3.

**FIGURE 4:**
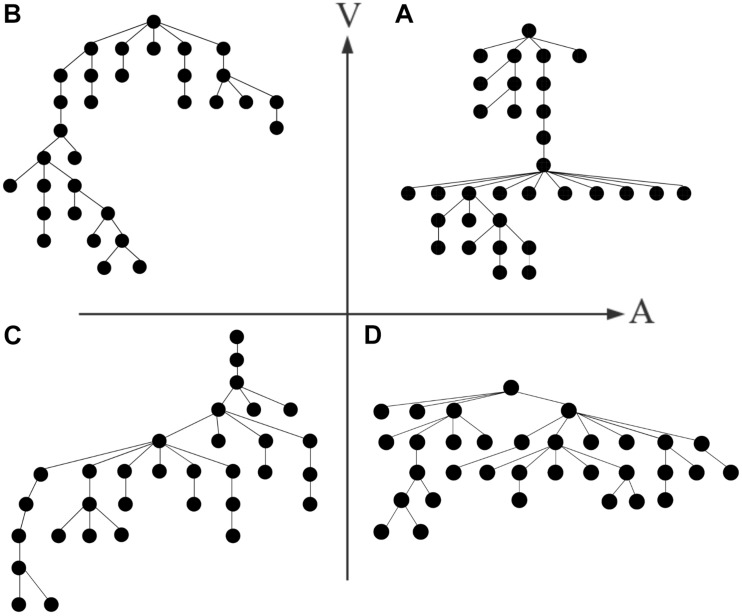
Corresponding to Figure 3, hierarchical structures of the MST brain networks of HAHV **(A)**, LAHV **(B)**, LALV **(C)**, and HALV **(D)** in the γ band.

For the same emotion, the minimum spanning trees of different subjects overlapped to form a connected graph under the condition that the same node exists. The weight of the edge in a connected graph means the number of overlapping edges of minimum spanning trees. An MST of the connected graph generated by the Kruskal algorithm is an average minimum spanning tree (average MST) of this emotion. Figure 3 shows the topological structure of the average MST for the four types of emotions in the γ band, in which the average MST edges mean the edges of maximum weights in the connected graph. Figure 4 shows the tree hierarchy of the MST corresponding to Figure 3 for each of the four different emotions in the γ band.

We repeatedly applied an analysis of variance measurement in the 2 (arousal/valence) × 2 (high/low) within-subject experimental region. The frequency ranges with significant differences were mainly in the γ band (Table 2). It was found that there were significant differences in MPLI characteristics under arousal conditions [*F*(29,1) = 47.8, *P* = 0.000, ES = 0.614], valence conditions [*F*(29,1) = 6.548, *P* = 0.016, ES = 0.179] and at interactions between the valence and arousal conditions [*F*(29,1) = 5.015, *P* = 0.033, ES = 0.143]. Additionally, there were significant differences in Degree_max_ under arousal conditions [*F*(29,1) = 58.569, *P* = 0.000, ES = 0.611], valence conditions [*F*(29,1) = 29.718, *P* = 0.000, ES = 0.498] and the interaction between the valence and arousal conditions [*F*(29,1) = 4.423, *P* = 0.044, ES = 0.128]. Furthermore, there were significant differences in leaf fraction under arousal conditions [*F*(29,1) = 29.169, *P* = 0.000, ES = 0.493], valence conditions [*F*(29,1) = 7.48, *P* = 0.01, ES = 0.2] and the interaction between valence and arousal conditions [*F*(29,1) = 4.443, *P* = 0.043, ES = 0.129]. Meanwhile, there are also significant differences in the diameter and eccentricity of the minimum spanning tree. Diameter characteristics are given for arousal conditions [*F*(29,1) = 67.076, *P* = 0.000, ES = 0.691], valence conditions [*F*(29,1) = 14.23, *P* = 0.001, ES = 0.322] and for the interaction between valence and arousal conditions [*F*(29,1) = 4.478, *P* = 0.043, ES = 0.13]. Eccentricity characteristics are given for arousal conditions [*F*(29,1) = 63.574, *P* = 0.000, ES = 0.679], valence conditions [*F*(29,1) = 15.937, *P* = 0.000, ES = 0.347] and the interaction between valence and arousal conditions [*F*(29,1) = 5.213, *P* = 0.03, ES = 0.148].

**TABLE 2:** The interaction in the γ band between arousal and valence of repeated measurement analysis of variance.

**Within-subjects effects characteristic**	***F*(29,1)**	***P***	**ES**
**Arousal × Valence**			
MPLI	5.015	0.033	0.614
Degree_max_	4.423	0.044	0.128
BC_max_	4.065	0.053	0.119
Leaf fraction	4.443	0.043	0.129
Diameter	4.478	0.043	0.13
Eccentricity	5.213	0.03	0.148
tree Hierarchy	0.008	0.93	0.0002

In order to explore the interaction between valence and arousal, a paired *t*-test and an FDR correction was carried out for the characteristic values with significant differences in interaction between valence and arousal.

### Statistical Analysis Results for HAHV and LAHV

**FIGURE 5:**
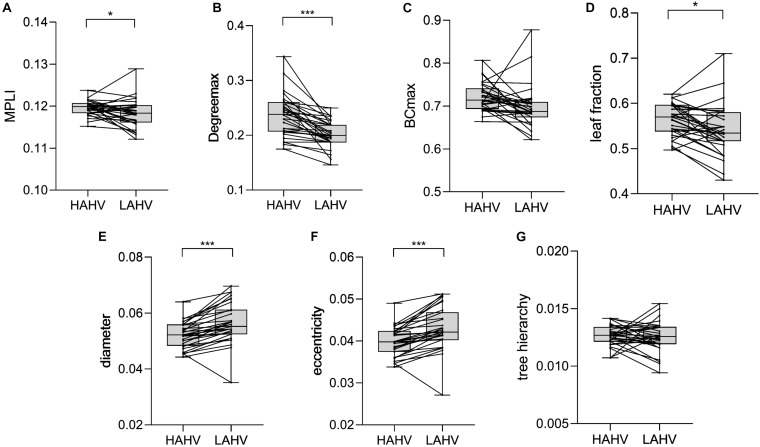
Changes in the value of MPLI **(A)**, Degree_max_ **(B)**, BC_max_ **(C)**, leaf fraction **(D)**, diameter **(E)**, eccentricity **(F)** and tree hierarchy **(G)** between HAHV and LAHV in the γ band. Significant values are indicated with asterisks: *P < 0.05, ***P < 0.001.

The comparison results show that the MST characteristics of Degree_max_ (*P* = 0.000, *t* = 6.241), leaf fraction (*P* = 0.016, *t* = 2.594), diameter (*P* = 0.000, *t* = −5.584), eccentricity (*P* = 0.000, *t* = −5.609) and MPLI (*P* = 0.036, *t* = 2.326) had significant differences (Table 3). The values of MPLI, Degree_max_ and leaf fraction were higher for HAHV than for LAHV, but the values of eccentricity and diameter were lower for HAHV than for LAHV (Figure 5).

**TABLE 3:** After paired *t* test and FDR correction, significant changes in MPLI and MST characteristics are seen between emotions in the γ band.

**Characteristic**	**Emotion**	**Mean ± SD**		***t***	***P***
MPLI	HAHV	0.119 ± 0.0017	HAHV-LAHV	2.326	0.036
	LAHV	0.118 ± 0.0033	HALV-LALV	5.572	0
	HALV	0.121 ± 0.0027	HAHV-HALV	–4.752	0
	LALV	0.118 ± 0.0032	LAHV-LALV	–0.175	0.862
Degree_max_	HAHV	0.236 ± 0.037	HAHV-LAHV	6.227	0
	LAHV	0.201 ± 0.024	HALV-LALV	5.903	0
	HALV	0.27 ± 0.052	HAHV-HALV	–4.467	0
	LALV	0.214 ± 0.036	LAHV-LALV	–2.345	0.026
Leaf fraction	HAHV	0.562 ± 0.035	HAHV-LAHV	2.658	0.016
	LAHV	0.54 ± 0.056	HALV-LALV	5.218	0
	HALV	0.593 ± 0.052	HAHV-HALV	–3.537	0.002
	LALV	0.546 ± 0.048	LAHV-LALV	–0.671	0.507
Diameter	HAHV	0.052 ± 0.0045	HAHV-LAHV	–5.584	0
	LAHV	0.056 ± 0.0071	HALV-LALV	–6.942	0
	HALV	0.048 ± 0.0045	HAHV-HALV	7.448	0
	LALV	0.055 ± 0.0062	LAHV-LALV	1.048	0.303
Eccentricity	HAHV	0.039 ± 0.0034	HAHV-LAHV	–5.609	0
	LAHV	0.042 ± 0.0051	HALV-LALV	–6.848	0
	HALV	0.037 ± 0.0033	HAHV-HALV	8.096	0
	LALV	0.042 ± 0.0048	LAHV-LALV	1.148	0.26

### Statistical Analysis Results for HALV and LALV

**FIGURE 6:**
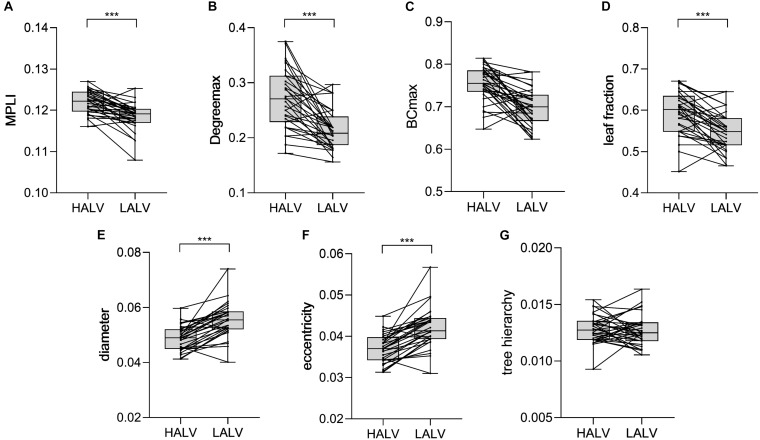
Changes in the value of MPLI **(A)**, Degree_max_ **(B)**, BC_max_ **(C)**, leaf fraction **(D)**, diameter **(E)**, eccentricity **(F)** and tree hierarchy **(G)** between HALV and LALV in theγband. Significant values are indicated with asterisks: ***P < 0.001.

The comparison results showed that there were significant differences in the MST characteristics (Table 3). Degree_max_ (*P* = 0.000, *t* = 5.903), leaf fraction (*P* = 0.001, *t* = 5.218), diameter (*P* = 0.000, *t* = −6.942), eccentricity (*P* = 0.000, *t* = −6.848) and MPLI (*P* = 0.000, *t* = 5.572). The MPLI, Degree_max_ and leaf fraction values were higher for HALV than for LALV, but the eccentricity and diameter values were lower for HALV than for LALV (Figure 6).

### Statistical Analysis Results for HAHV and HALV

**FIGURE 7:**
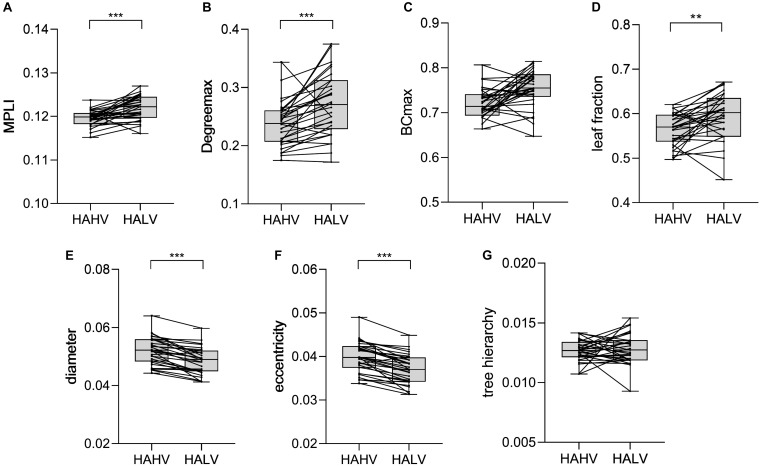
Changes in the value of MPLI **(A)**, Degree_max_ **(B)**, BC_max_ **(C)**, leaf fraction **(D)**, diameter **(E)**, eccentricity **(F)** and tree hierarchy **(G)** between HAHV and HALV in the γ band. Significant values are indicated with asterisks: **P < 0.01, ***P < 0.001.

The comparison results show that the MST characteristics Degree_max_ (*P* = 0.000, *t* = −4.467), leaf fraction (*P* = 0.002, *t* = −3.537), diameter (*P* = 0.000, *t* = 7.448), eccentricity (*P* = 0.000, *t* = 8.096) and MPLI (*P* = 0.000, *t* = −4.752) had significant differences (Table 3). The MPLI, Degree_max_ and leaf fraction values for HALV were higher than for HAHV, but the eccentricity and diameter values for HALV were lower than for HAHV (Figure 7).

### Statistical Analysis Results for LAHV and LALV

**FIGURE 8:**
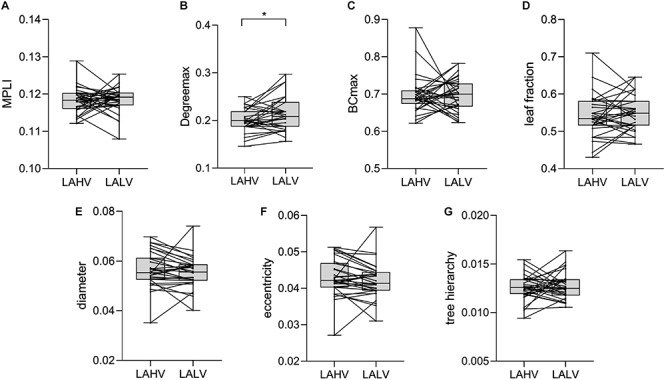
Changes in the value of MPLI **(A)**, Degree_max_ **(B)**, BC_max_ **(C)**, leaf fraction **(D)**, diameter **(E)**, eccentricity **(F)** and tree hierarchy **(G)** between LAHV and LALV in the γ band. Significant values are indicated with asterisks: *P < 0.05.

The comparison results show that the MST characteristic Degree_max_ (*P* = 0.026, *t* = −2.367) had significant differences (Table 3). The comparison results of LAHV and LALV in the γ band show that the Degree_max_ of LALV were larger than those of LAHV (Figure 8).

### The Distribution of Hub Nodes in the MST

Some interesting findings emerged from studying the topology of the MST. The topological structure of the mean MST represents the structure of the minimum spanning tree of the brain network of most subjects under a certain emotion. The large node degrees in an average MST represent that most of the minimum spanning trees have connections in these nodes, which can reflect whether the brain network is active (Table 4). In the γ band, the hub nodes of the MSTs for different emotions were distributed across different brain regions (Figure 3). In the MST of HAHV, the hub nodes were mainly concentrated in the frontal lobe area (FP1, FC5) and temporal lobe (T7, T8). The hub nodes in the MST of HALV were mainly concentrated in the frontal lobe (FP2), occipital area (O2) and temporal lobe (T8). The hub nodes in the MST of LAHV were mainly concentrated in the frontal area (FP1, FC5). The hub nodes in the MST of LALV were mainly concentrated in the central parietal (CP5, CP6) and temporal lobe areas (T7).

**TABLE 4:** Nodes with an average minimum spanning tree degree in the γ band greater than or equal to 3.

**HAHV**	**HALV**	**LAHV**	**LALV**
T7 (*k* = 9)	FP2 (*k* = 5)	FP1 (*k* = 4)	FC5 (*k* = 7)
FC5 (*k* = 5)	T8 (*k* = 5)	FC5 (*k* = 4)	T7 (*k* = 5)
FP1 (*k* = 4)	O2 (*k* = 4)	CP6 (*k* = 4)	FP1 (*k* = 4)
T8 (*k* = 4)	FP1 (*k* = 3)	F3 (*k* = 3)	CP5 (*k* = 4)
FP2 (*k* = 3)	FC5 (*k* = 3)	T7 (*k* = 3)	CP6 (*k* = 4)
Fz (*k* = 3)	FC6 (*k* = 3)	F4 (*k* = 3)	Pz (*k* = 3)
	P8 (*k* = 3)	F8 (*k* = 3)	P8 (*k* = 3)
		C4 (*k* = 3)	

## Discussion

There are significant differences in network characteristics between HAHV and LAHV, HALV and LALV, and HAHV and HALV in the γ band. In other recent research related to emotion, significant differences were also mainly concentrated in the γ band (Mu et al., 2017; Batashvili et al., 2019). In addition, recent studies on emotion classifiers have found that the γ band provides the highest classification accuracy of any frequency band (Mohammadi et al., 2017).

Many studies have also found that the frontal, temporal, parietal and occipital areas of the brain are related to emotional processing from the perspectives of MRI and EEG (Guntekin and Basar, 2007; Ma et al., 2012; Aydin et al., 2018; Zheng et al., 2019). This concept is consistent with our research showing that the hub nodes of the MST are concentrated in the frontal, temporal, parietal and occipital areas.

Under the same high valence level, HAHV showed a significant higher MPLI than LAHV. MPLI represents the average connection strength of different brain regions during different emotional stimuli. The average connection strength of the brain network is greater for HAHV than for LAHV, implying that, under the same high valence of stimulation, high arousal can stimulate greater brain activity than low arousal. HAHV showed a significant higher leaf fraction than LAHV, while HAHV showed significant lower diameter and eccentricity than LAHV. A large leaf fraction indicates that many leaf nodes exist in the MST network, and the network tends to have a star-shaped topology. In networks with many leaf nodes, the key routes of information flow converge at one or a few hub nodes. Therefore, the BC_max_ and Degree_max_ values of these hub nodes are large. Our results are consistent with the above: HAHV showed significant higher Degree_max_ and leaf fraction than LAHV. Eccentricity is a local node characteristic. Nodes with low eccentricity are central nodes. The smaller the eccentricity value of a node, the closer the node is to the central node location. When the average eccentricity of an MST is small, the topological structure of the MST tends to be star-shaped. Diameter is the longest path between any two nodes in the MST. A decrease in diameter means that the number of leaves increases, which further shows that the topology of the MST tends to be star-shaped. The star topological structure corresponds to a random brain network (Figure 2). Consequently, the MST topology of HAHV showed more star-shaped configuration than that of LAHV, which indicates that a high-arousal network has more random connections than a low-arousal network. A study by Varotto found that in normal subjects, pleasant music caused an increase in the number of brain network connections compared to a resting state (Varotto et al., 2012). In other words, their results are consistent with ours.

Under the same low valence level, HALV showed a significant higher MPLI than LALV, indicating that the average connection strength of the brain is greater for HALV than for LALV. Under the same low valence, high arousal can stimulate the brain more actively than low arousal, which is consistent with the above conclusion. HALV showed significant higher Degree_max_ and leaf fraction than LALV, while HALV showed significant lower diameter and eccentricity than LALV. This result indicated that the MST topology of HALV showed a more star-shaped configuration than that of LALV. At this time, the values of BC_max_ and Degree_max_ of hub nodes also increased with arousal. As confirmed by our results, HALV showed a significant higher Degree_max_ than LALV. Meanwhile, HALV showed significant lower eccentricity and diameter than LALV, which further shows that the topological structure of HALV is closer to a star configuration. Compared to the brain network associated with LALV, the increase toward randomness of brain network associated with HALV can be proven. In general, a high-arousal brain network has more random connections than does a low-arousal brain network. Nicola Martini et al. also found a difference in phase synchronization between unpleasant and neutral stimuli in the γ band (Martini et al., 2012). The studies of Balconi and Lucchiari showed that compared with low arousal (sadness), high arousal (anger and fear) increased the brain’s γ activity (Balconi and Lucchiari, 2008). Miskovic found that EEG coherence increased when subjects viewed high-arousal pictures (Miskovic and Schmidt, 2010).

Chanel found that the emotion of valence is more difficult to recognize than that of arousal, which supports the view that the correlation between physiological signals and arousal is better than that of valence (Chanel et al., 2009). As discussed earlier, in the comparison of arousal, there were significant differences among different levels of arousal in the same valence level. However, when compared with different valence aspects at the same arousal level, there was little significant difference in characteristics between LAHV and LALV. A possible explanation is that the low arousal and emotional activation of LAHV and LALV resulted in no significant characteristic difference.

Interestingly, there were significant differences in MPLI and MST characteristics between HAHV and HALV. Initially, HALV showed a significant higher MPLI than HAHV and it indicated the average connection strength of the brain network was greater in HALV than in HAHV. Brain activation is higher and more information is transmitted between brain regions in a low-valence emotion than in a high-valence emotion. The brain response to HALV (anger and shocked) stimulation is stronger compared with HAHV (happy and excited) stimulation. Meanwhile, HALV showed significant higher Degree_max_ and leaf fraction than HAHV and significant lower diameter and eccentricity than HAHV. This result indicated that the MST topology of HALV showed more star-shaped configuration than that of HAHV. HAHV showed significant higher eccentricity and diameter than HALV, which further illustrated that the MST topology of HAHV showed a more line-shaped configuration than that of HALV. From the perspective of a brain network, there are more random connections between brain regions and the development of the brain network structure tends toward randomization in a HAHV brain network compared to a HALV brain network. Consistent with our findings, Ma also found that the characteristic path length of the brain network for negative emotions is longer than that of positive emotions. A short characteristic path represents random connection increases in the brain network. That means when the brain deals with negative emotions, brain regions are more activated (Ma et al., 2012).

## Conclusion

The results show that there are significant differences in MPLI and MST characteristics among different emotions in the γ band. From the perspective of the MST, high-arousal trees showed a more star-shaped configuration than low-arousal trees. However, the low-valence tree showed a more star-shaped configuration than the high-valence tree in the same high arousal level. This result indicated that the brain networks for low-valence states had a more random topological structure and more connections between brain regions than the brain networks of high-valence states. This study provides theoretical support for research on negative bias.

## Data Availability Statement

The datasets for this study can be found in the DEAP (http://www.eecs.qmul.ac.uk/mmv/datasets/deap/).

## Ethics Statement

Ethical review and approval was not required for the study on human participants in accordance with the local legislation and institutional requirements. The patients/participants provided their written informed consent to participate in this study.

## Author Contributions

RC designed the research and developed the method. YH analyzed the data with the support of HS and SH. YH wrote the first draft of the manuscript, which all authors revised and approved. XW and YG provide help in algorithms and programs, as well as in the use of professional software. RC directed the study. All authors participated to the scientific discussion.

## Conflict of Interest

The authors declare that the research was conducted in the absence of any commercial or financial relationships that could be construed as a potential conflict of interest.
